# Responsible AI for Predicting Delayed Hospital Discharge Among Older Adults: Development and Evaluation Study for Balancing Accuracy, Equity, and Explainability

**DOI:** 10.2196/83244

**Published:** 2026-04-13

**Authors:** Somayeh Ghazalbash, Manaf Zargoush, Sara JT Guilcher, Kerry Kuluski

**Affiliations:** 1 Health Policy and Management DeGroote School of Business McMaster University Hamilton, ON Canada; 2 Leslie Dan Faculty of Pharmacy University of Toronto Toronto, ON Canada; 3 Department of Physical Therapy Temerty Faculty of Medicine University of Toronto Toronto, ON Canada; 4 Institute for Better Health, Trillium Health Partners Mississauga, ON Canada; 5 Institute of Health Policy, Management, and Evaluation University of Toronto Toronto, ON Canada

**Keywords:** delayed discharge, responsible AI, explainability, equity, algorithmic fairness, machine learning, responsible artificial intelligence

## Abstract

**Background:**

Amid growing demands and constrained health care resources, effective hospital bed capacity management is crucial. Delayed hospital discharge, where patients remain in the hospital beyond the need for acute care, strains resources, affects patient outcomes, and reduces system efficiency. Predicting such delays facilitates early interventions to avert them and alleviate burdens on patients, care partners, hospitals, and the broader health care system.

**Objective:**

This study aimed to develop comprehensive predictive analytics for delayed discharges among older adults using explainable machine learning to boost transparency and interpretability, while integrating fairness to mitigate algorithmic biases.

**Methods:**

Leveraging longitudinal data from over 2 decades in Ontario, Canada, we applied extreme gradient boosting and logistic regression models to predict delayed discharges within 90 days post–acute care. Data preprocessing included a 2-year look-back for clinical histories and balanced sampling to address class imbalance. Model performance was assessed via area under the receiver operating characteristic curve, calibration, and clinical utility. Fairness was evaluated across sex, urban or rural residence, and residential instability using several threshold-free metrics. Explainability was examined at the global model level (via partial dependence plots and permutation feature importance) and locally (via Shapley Additive Explanations, breakdown, and ceteris paribus methods), with principal component analysis used to cluster key features for high-risk patients.

**Results:**

The extreme gradient boosting model outperformed logistic regression, achieving an area under the receiver operating characteristic curve of 0.82 on the test set, with acceptable within-group and cross-group ranking fairness across subgroups. Explainability clustering analyses identified functional and cognitive declines (eg, care support needs, dementia, and mobility issues) and regional disparities as primary drivers of high-risk predictions. Bias mitigation improved calibration parity, especially when stratifying by residential instability, underscoring the trade-offs policymakers must weigh between accuracy, fairness, and explainability.

**Conclusions:**

This study demonstrates the potential of responsible artificial intelligence in health care, emphasizing the need to balance predictive accuracy, equity, and interpretability. It uncovers systemic gaps and offers actionable insights for enhanced discharge planning, resource optimization, and equitable care delivery.

## Introduction

Delayed hospital discharge (DHD) is defined as staying in hospitals longer than medically necessary. It has become a critical issue due to increasing health care demands under constrained and limited resources. DHD is a prevalent issue in health care systems worldwide, affecting numerous countries, including Canada, the United States, the United Kingdom, Germany, Norway, Sweden, Scotland, Denmark, France, New Zealand, Australia, and South Korea [1]. These delays often stem from unavailable postacute options needed to ensure patients’ safe discharge, such as long-term care (LTC), rehabilitation, or other community support services [2,3]. DHD is more common among older adults, especially those with complex health needs due to frailty and multiple chronic conditions [4].

DHD has both direct and indirect impacts on the patients. DHD adversely affects patients and their care partners directly through increased risk of adverse health outcomes, including functional decline, evidenced by a 3%-5% functional deterioration for each day of inactivity [5]. DHD status is also associated with a higher risk of hospital harm and mortality [2]. Patients and care partners often report increased stress, emotional strain, and uncertainty regarding care planning, further compounding the burden of DHD [6].

DHD also has negative effects on the overall efficiency of the health care system. In 2022-2023, DHD patients accounted for 17% of all acute-care bed-days in Canada, with provincial rates ranging from 6.8% to 26% [7]. The occupancy levels of acute-care beds by patients who no longer need such care contribute to system-wide inefficiencies, such as delayed admissions, overcrowding in emergency departments, surgical cancellations, and a domino effect on the entire health care system [2,5,8], leading to hallway medicine [9]. The financial burden associated with DHD is substantial, with hospital-based care for these patients estimated at CAD $730 (US $524.68) to CAD $1200 (US $862.48) per day, significantly exceeding the costs of alternative settings such as LTC (CAD $126 [US $90.56]-CAD $144 [103.50]/day), transitional care units (CAD $155 [US $111.40]/day), or home-based services (CAD $42 [US $30.19]-CAD $118 [US $84.81]/day) [7]. In Canada, inefficiencies related to DHD are estimated to cost the health care system between CAD $5 million (US $3.59 million) and CAD $9 million (US $6.47 million) daily, amounting to billions of dollars annually [10]. Older adults are disproportionately affected by DHD, particularly in Canada, where Ontario, the country’s most populous province, reported that over 80% of DHD designations in acute care involve adults aged 65 years and older, with 64% of them older than 75 years [11,12]. Although increasing system capacity, especially in LTC, has been proposed as a potential solution, this strategy requires substantial capital investment and is not practical in the short or medium term [13] and does not address the underlying issues of an undersupply of housing with care models (such as supportive housing or assisted living) and lack of chronic disease management in the community, which could mitigate unnecessary use of hospital care.

One promising alternative solution is to adopt a proactive approach. The risk of DHD can be predicted in advance, enabling health care providers to identify high-risk patients early and implement targeted interventions to reduce its occurrence. Machine learning (ML)–based methodologies are gaining popularity, especially in health care, due to their superior predictive accuracy compared to traditional statistical methods [14] and their effectiveness with longitudinal electronic health records [15,16]. Importantly, identifying a patient at elevated risk of DHD enables the development of upstream, community-based proactive models of care that aim to prevent the clinical and social deterioration leading to hospitalization with a discharge delay, rather than solely improving discharge planning after hospitalization has occurred. Such forward-looking models of care can integrate both health and social care resources, strengthening linkages to primary care and connecting patients and caregivers to essential community supports. These supports may include assistance with instrumental activities of daily living (eg, meal preparation and transportation to medical appointments) as well as fundamental activities of daily living (eg, mobility, eating, and bathing). By addressing these needs proactively, the care model is designed to stabilize older adults within the community, reduce avoidable hospitalizations, and ultimately minimize downstream discharge delays. Timely and accurate risk predictions, therefore, provide critical information for targeting preventive interventions, allocating resources more effectively, and improving patient flow across the continuum of care [17].

Although ML predictions are promising, they present a complex trade-off between accuracy, fairness, and explainability [18]. ML algorithms trained on historical data may unintentionally perpetuate biases [19], reinforcing existing social inequalities and disproportionately affecting marginalized populations [20]. Several studies have shown that automatic algorithms can systematically discriminate against equity-seeking groups, amplifying disparities rather than alleviating them [19,21]. This highlights the need to integrate fairness into predictive models to avoid unjustified disparities in model predictions across groups, thereby ensuring equitable outcomes.

Along with fairness, transparency and explainability are also crucial for ensuring trust and encouraging adoption in clinical settings. Health care professionals not only require accurate and fair predictions but also need clear, interpretable explanations to understand and validate the reasoning behind them, enabling them to integrate artificial intelligence (AI) insights into their decision-making [22]. This has driven the emergence of explainable artificial intelligence (XAI), which addresses concerns about algorithmic opacity by developing models that are both interpretable and accountable. XAI tackles AI’s “black box” nature by making predictions interpretable and transparent. This transparency is crucial, especially in health care, where trust as well as shared and informed decision-making are vital for patients’ buy-in and optimizing clinical outcomes and promoting equitable care.

Most of the existing research on DHD has focused on identifying associated patient characteristics and strategies to improve patient flow [3,5,23,24]. Limited studies have investigated predictive upstream approaches to identify patients vulnerable to experiencing delayed discharge. Previous research has applied different methods, ranging from more traditional statistical models, such as logistic regression (LR) [23], to rule-based systems [25] and ML techniques, including neural networks [26]. Such efforts underscore the potential of predictive models to enhance care planning processes and inform decision-making. However, in reviewing the published literature to date, several key gaps emerge. First, the use of ML to predict delayed discharge with large and comprehensive datasets is limited, which impacts the robustness and accuracy of existing models. Second, there is a notable lack of emphasis on responsible AI principles within these models. Addressing these gaps is vital for developing reliable and actionable strategies to effectively prevent and manage delayed discharge. To address these challenges, our study aimed to contribute to the existing literature by (1) developing ML-based predictive models for identifying patients at risk of DHD among older adults, (2) examining the fairness implications of the developed ML-based predictive models to ensure outcome parity among equity-seeking groups, and (3) enhancing the interpretability and explainability of the ML-based predictive models by applying XAI at both global (population-specific) and local (person-specific) levels.

## Methods

### Ethical Considerations

This study received approval from the HiREB (Hamilton Integrated Research Ethics Board) in Ontario (Review Reference: 2025-5472-AP). This study used record-level, coded, and linkable administrative health data held at ICES (Institute for Clinical Evaluative Sciences). Individual informed consent was not required because the study involved the linking together of historical records of personal health information, and consent was impracticable or impossible to obtain. At ICES, all data collections are subject to rigorous privacy and security controls. Participants received no compensation.

### Proposed Methodology

**Figure 1:**
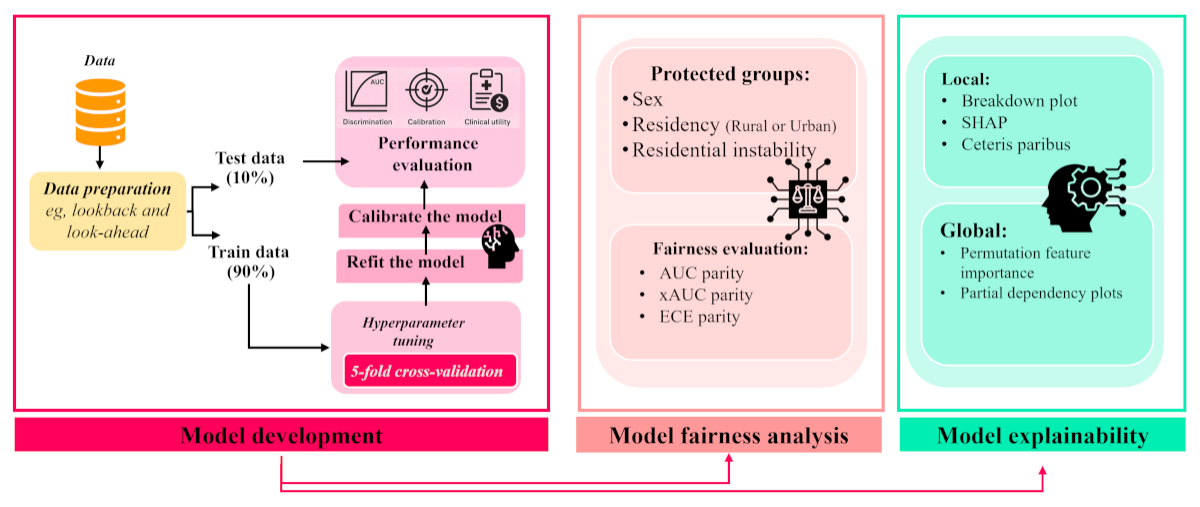
Study methodology framework. AUC: area under the receiver operating characteristic curve; ECE: expected calibration error; SHAP: Shapley Additive Explanation; xAUC: cross-group area under the curve.

We developed a 3-step methodology for this research (Figure 1). The first step involved preparing the data and fine-tuning hyperparameters for the ML algorithms to identify the best prediction model. We used various measures of predictive accuracy to evaluate the model’s performance (model development step) and then assessed algorithmic bias across protected groups using multiple fairness metrics (model fairness analysis step). Finally, we examined the model’s explainability from both local and global perspectives and used clustering techniques to find important predictive features (model explainability step).

### Study Population

A retrospective, observational cohort study was conducted using 2 secondary administrative health databases housed at the ICES, including the discharge abstract database and registered persons database. These databases were linked through a unique patient identifier. The cohort included Ontario acute-care admissions from 2004 to 2022, including approximately 1.8 million patients with nearly 12 million observations. Individuals were excluded from the cohort if they were younger than 65 years of age, non-Ontario residents, had missing or invalid data, or were admitted from LTC facilities, resulting in a total of 9 million observations with 1.3 million patients. We excluded admissions from LTC due to their distinct care pathways and discharge patterns. Patients from LTC typically return to their homes after shorter stays, influenced by policies that protect their LTC beds. Including them would obscure the patterns of discharge delays and community transitions central to our research, which aims to support aging in the community.

All variables, including predictors and outcomes, were derived from structured, coded administrative data at ICES, with no manual chart abstraction or unstructured text processing. More detailed information regarding the data source is available in Multimedia Appendix 1.

### Data Descriptions

The choice of candidate predictors was based on prior research and data availability, including (1) demographic factors, such as sex and age; (2) socioeconomic factors, including the Ontario Marginalization Index (ON-Marg 2016) [27], income quintile, and rural or urban residency; (3) geographic factors, such as regions; and (4) clinical factors, including patient comorbidities, palliative care, home care, rehabilitation care, fracture, fall, mobility, and disabilities. The primary outcome was DHD within 90 days, reflecting a clinically relevant transition period from the hospital to the next point of care. In Canada, the term DHD refers to the alternate level of care designation, a classification system used in hospitals to identify inpatients who no longer require the full intensity of acute medical services but are not yet ready to be safely discharged home. A patient must be designated as DHD by the most suitable member of the care team, such as a physician, LTC assessor, patient care manager, or discharge planner. The determination of DHD status is a clinical responsibility, based on various indicators of patient readiness and ongoing care needs. These indicators include clinical stability, safety risks, activity tolerance, medication or fluid requirements, diagnostic and therapeutic needs, as well as considerations related to mental health, respiratory care, or palliative care. The DHD designation is formally coded and routinely collected in Canadian hospital administrative data [28]. More detailed information regarding variable selection for model building is also given in Multimedia Appendix 1. To ensure the robustness of our findings, sensitivity analyses were conducted using alternative timeframes of 180 and 365 days, with the results presented in Multimedia Appendix 2.

### Data Preprocessing and Preparation

We used a 2-year look-back window to capture patients’ clinical history, including prior comorbidities. Further details regarding the construction of these variables can be found in Multimedia Appendix 2. We dichotomized the socioeconomic variables from the ON-Marg (including ethnic concentration, dependency, residential instability, and material deprivation) [27] into binary variables, comparing the most marginalized quintile (Q5) with the other quintiles (Q1-Q4). Missing data were rare (overall <0.3%) and were primarily attributable to incomplete values in the ON-Marg socioeconomic indices. Given the negligible degree of missingness and to avoid introducing additional assumptions through imputation, observations with missing values were removed using a complete-case approach.

### Predictive Models

We used supervised ML to predict DHD risk with the extreme gradient boosting (XGB) algorithm. We chose this algorithm due to its superior performance with complex datasets, its ability to generalize, and its robustness against noise and outliers [29,30]. In addition to this ML algorithm, we also included LR as a traditional predictive approach in health care, due to its explanatory power and widespread use. By including LR alongside XGB, we aimed to compare its predictive performance with that of more advanced ML techniques and evaluate the suitability of a widely used model for our specific prediction task. We established the models using the same data structure, preparation, training, and assessment protocols to ensure a fair and meaningful comparison.

Following the standard practice, we used a 2-stage evaluation approach. First, the dataset was split at the patient level into training (90%) and testing (10%) sets to prevent information leakage from repeated visits. The test set, which remained entirely separate from the training process, was exclusively used for the final evaluation of the model’s predictive performance. In the second stage, we used 5-fold cross-validation within the training set to optimize hyperparameters and mitigate the risk of overfitting. After the optimal hyperparameters were identified, the model was retrained from scratch on the entire training set. The resulting model was evaluated on the untouched 10% test set, yielding the reported performance metrics. To ensure that the predicted readmission probabilities accurately reflected the true underlying risk, we performed post hoc calibration of our predictive models. Calibration measures the agreement between the predicted probability and the observed (empirical) probability of the outcome class. As the true probability is unknown, proxies such as the proportion of outcomes of interest within a specific group of observations are often used instead of the observed probability [31]. We applied Platt scaling (also known as logistic calibration and sigmoid calibration), a method that transforms model estimates using a trained sigmoid function. It is a univariate LR model with model estimates as independent variables and binary outcomes as dependent variables [32]. The complete pipeline is illustrated in Figure 1.

To ensure a comprehensive and clinically actionable assessment, each predictive model was evaluated across 3 key dimensions: discrimination, calibration, and clinical utility. Discrimination was quantified using the area under the receiver operating characteristic curve (AUC), which measures the model’s ability to distinguish patients with and without the outcome. Calibration was assessed using the Brier score and the expected calibration error (ECE), with reliability diagrams used to visualize agreement between predicted probabilities and observed outcomes [33]. The clinical utility was evaluated through decision curve analysis (DCA), which quantifies the clinical net benefit (NB) of our predictive models across various risk thresholds [34]. The NB is calculated as the expected benefit to the cases (ie, true positive rate) minus the expected harm to the controls (ie, false positive rate multiplied by the threshold probability). For the sake of clarity, we used the standard NB, which has a maximum value of 1.0 [35]. In the DCA, our predictive models are compared against two extreme clinical strategies: (1) treating all patients as positive cases, known as the “treat all” strategy, and (2) treating all patients as negative cases, referred to as “treat none” [36]. In the former scenario, the true positive rate is equal to the rate of the high-risk event, π, and the false positive rate is equal to 1–π. In the latter scenario, the NB is equal to zero [37]. Additionally, we conducted a temporal validation to further evaluate the robustness and external validity of our predictive model under realistic deployment conditions. We partitioned the cohort at the patient level based on the year of each patient’s first recorded admission, assigning those with initial admissions before 2014 to the training set and those with initial admissions from 2015 onward to the temporal test set. Data preprocessing, ML model development, and evaluations were performed using R (version 4.2.2; R Foundation). All details regarding the software packages used for each analysis are provided in Multimedia Appendix 3.

### Fairness Assessment

We calculated multiple threshold-free fairness metrics for each model to evaluate algorithmic fairness. We used threshold-free fairness metrics to better reflect the application of predictive models in health care, where continuous risk probabilities inform patient prioritization and resource allocation rather than rigid binary classifications. Essentially, in health care settings, risk scores inform care intensity and preventive interventions as ranking tools rather than binary classifiers. Thus, threshold-free measures provide better insights into disparities in utility and resource allocation than threshold-dependent criteria. We evaluated within-group and cross-group ranking fairness using AUC and xAUC (cross-group area under the curve) parity [38], respectively, and examined calibration fairness using ECE parity [39]. AUC parity evaluates the consistency of within-group discriminatory performance, comparing how well each group’s positive cases are distinguished from its negatives. xAUC parity measures disparities in misranking errors caused by a predictive score. It quantifies the difference in the probabilities of ranking a random positive case from 1 protected group above a negative one from another group, and vice versa [40]. ECE parity assesses whether a model’s predicted probabilities are equally well calibrated across regular and equity-seeking groups [39].

Certain socioeconomic groups, such as females or racial minorities, are often underrepresented in health care data because of biological or nonbiological variation [41,42]. Rural residency is recognized as a distinct barrier to care access, leading to potentially vulnerable or underserved subpopulations [43]. Similarly, residential instability reflects the underlying social vulnerabilities, such as housing insecurity and social isolation [27]. These disparities can introduce or amplify biases in ML predictions and decisions, potentially compromising the quality of care delivered to these equity-seeking groups [44]. To address this, we considered 3 equity-seeking groups in this study: females (vs males), rural (vs urban), and highly marginalized (vs low- to moderate-marginalized) in terms of residential instability. A summary of the prevalence of these equity-seeking groups across the training and test data is provided in Multimedia Appendix 1.

The relative fairness metrics are measured as the ratio of equity measures for the equity-seeking group to those for the regular group in the constructed predictive models. Therefore, a ratio of 1.0 indicates perfect fairness, with any deviation (above or below 1.0) reflecting unfairness toward regular or equity-seeking groups. Finally, to reduce potential algorithmic bias in our model, we used a postprocessing bias mitigation approach based on group-fairness calibration refinement [38]. Separate Platt scaling models were fitted to the predicted probabilities within each subgroup defined by the equity-seeking attributes, ensuring equitable alignment of predicted risks with observed outcomes across groups.

### Model Explainability: Local and Global Perspectives

In contrast to traditional models such as LR, ML algorithms often lack interpretability, which makes it difficult to understand how predictors affect outcomes [45]. To tackle this challenge, we applied XAI principles to gain insights into both global and local (ie, patient-specific) explainability. Global explainability, which examines the overall association between predictors and the outcome, was explored using partial dependence plots (PDPs) and permutation feature importance [46]. While PDPs visualize the marginal effects of features on predictions, the permutation feature importance method quantifies how much a model’s performance changes when the impact of a feature is removed through resampling or permutation.

Local explainability, which focuses on understanding model predictions for individual cases, was achieved using SHAP (Shapley Additive Explanations) [47], breakdown [48], and ceteris paribus [46]. The SHAP method uses a game-theoretic approach to assess the contribution of each feature to a specific prediction by evaluating all possible combinations of features. The breakdown approach decomposes individual model predictions into interpretable components, attributing the final prediction to the cumulative contributions of specific features. These 2 methods are complementary. Breakdown plots are order-dependent, meaning the importance of features can vary with their sequence, and can be sensitive to feature correlations. In contrast, SHAP provides order-invariant, theoretically consistent attributions, though it does not visualize the incremental path from baseline to final prediction. By using both, we enable cross-validation of local explanations, mitigate order-induced artifacts, and provide a more robust and transparent interpretation of individual-level model decisions [46]. The ceteris paribus approach examines the effect of an explanatory variable while holding the values of all other variables constant. The primary aim is to understand how variations in the values of a variable influence the model’s predictions. We conducted the local explanation on a subsample (10%) of test data due to computational constraints for individual predictions.

Local explainability methods generate detailed, instance-specific insights into feature contributions, but analyzing these across numerous predictions can yield a high-dimensional dataset of metrics, including contributions (quantifying how much each feature influences the model’s prediction for a given patient), frequencies (capturing how often a particular factor appears among the top 10 features for high-risk patients, thereby indicating its prevalence across such cases), and rankings (denoting the order of importance for each feature, where a lower rank reflects greater consistent influence on predicting risk), which may obscure broader patterns. To address this, we applied principal component analysis (PCA) [49], a well-established unsupervised dimensionality reduction technique that identifies orthogonal components maximizing variance in the data, thereby uncovering latent structures and reducing complexity while preserving key information.

To construct the input dataset for PCA, we first aggregated all local explanation outputs for patients classified as high-risk (predicted probability greater than 0.5). For each predictor, we calculated three summary metrics reflecting its behavior across high-risk individuals: (1) average contribution to the prediction, (2) average rank based on its relative importance, and (3) the prevalence (percentage of high-risk patients for whom the feature appeared among the top contributors). All metrics were standardized using *z* scores to ensure equal weighting across variables. PCA was then applied to the resulting feature-by-metric matrix to reduce dimensionality and identify underlying latent structures. For interpretability, features were positioned within 1 of 4 quadrants (Q1-Q4) according to the signs of their PC1 and PC2 coordinates. To improve conceptual interpretation of the resulting clusters, each feature was categorized into 1 of 3 a priori groups: sociodemographic and geographic characteristics, multimorbidity and health status, and mobility and disability factors. These groups were then examined within each PCA quadrant to identify coherent patterns and clinically meaningful clusters. Additional details about this mapping can be found in Multimedia Appendix 4.

## Results

### Descriptive Results

Table 1 presents the distribution of sociodemographic, geographical, and clinical characteristics among patients with and without delayed discharge. DHD patients were more likely to be older (median age 84, IQR 79-89 vs 80, 74-85 years), female (201,015/793,276, 57% vs 3,911,541/8,107,313, 48%), and to have lower income (201,015/793,276, 25% in the lowest quintile vs 1,695,425/8,107,313, 21%). DHD patients were more likely to reside in more marginalized areas, particularly concerning residential instability (277,898/793,276, 35% vs 2,251,598/8,107,313, 28%), which reflects factors such as living alone or in nonowned dwellings. Additionally, they showed higher levels of dependency (319,730/793,276, 40% vs 2,993,423/8,107,313, 37%), indicating a greater proportion of seniors and individuals not engaged in the labor force. Furthermore, material deprivation was more prevalent among DHD patients (195,662/793,276, 25% vs 1,688,573/8,107,313, 21%), suggesting they face greater barriers to meeting basic needs such as employment and education [27]. More detailed results of the descriptive analysis of clinical features are provided in Multimedia Appendix 1.

**Table 1:** Patient sociodemographic and geographical characteristics.

Characteristic		Overall (N=8,900,589)	Without delayed discharge (N=8,107,313)	With delayed discharge (N=793,276)
**Region, n (%)**				
	Eastern	1,565,790 (18)	1,448,201 (18)	117,589 (15)
	Central	2,877,461 (32)	2,610,461 (32)	267,000 (34)
	Metropolitan	1,635,504 (18)	1,483,312 (18)	152,192 (19)
	Southwestern	1,908,596 (21)	1,748,197 (22)	160,399 (20)
	Northern	913,238 (10)	817,142 (10)	96,096 (12)
**Income level, n (%)**				
	Q1-lowest	1,896,440 (21	1,695,425 (21)	201,015 (25)
	Q2	1,895,832 (21)	1,722,811 (21)	173,021 (22)
	Q3	1,777,314 (20)	1,622,389 (20)	154,925 (20)
	Q4	1,665,924 (19)	1,529,430 (19)	136,494 (17)
	Q5-highest	1,665,079 (19)	1,537,258 (19)	127,821 (16)
**Residency, n (%)**				
	Urban	7,502,926 (84)	6,817,006 (84)	685,920 (86)
	Rural	1,397,663 (16)	1,290,307 (16)	107,356 (14)
**Sex, n (%)**				
	Female	4,362,152 (49)	3,911,541 (48)	450,611 (57)
	Male	4,538,437 (51)	4,195,772 (52)	342,665 (43)
Age (years), median (IQR)		80 (75-85)	80 (74-85)	84 (79-89)
ON-Marg^a^: ethnic concentration (Q5 vs others), n (%)		1,446,682 (16)	1,320,691 (16)	125,991 (16)
ON-Marg: dependency (Q5 vs others), n (%)		3,313,153 (37)	2,993,423 (37)	319,730 (40)
ON-Marg: residential instability (Q5 vs others), n (%)		2,529,496 (28)	2,251,598 (28)	277,898 (35)
ON-Marg: material deprivation (Q5 vs others), n (%)		1,884,235 (21)	1,688,573 (21)	195,662 (25)

^a^ON-Marg: Ontario Marginalization Index.

### Predictive Performance

**Figure 2:**
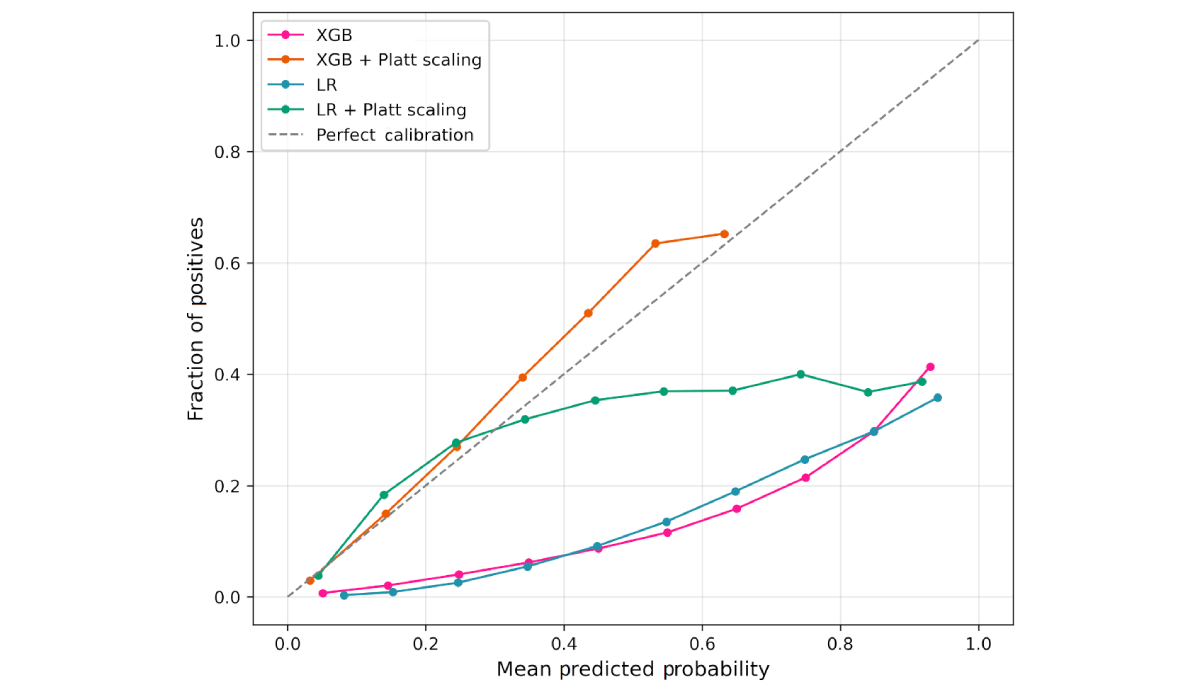
Reliability diagrams for XGB and LR models. LR: logistic regression; XGB: extreme gradient boosting.

Predictive models were evaluated using threshold-free performance metrics on the test set, as shown in Table 2. The results reveal that the XGB model achieved a slightly higher discrimination performance (AUC=0.822) compared to the LR model (AUC=0.799), indicating a modestly superior capacity to rank patients by risk. Both base models showed similar Brier scores (≈0.18), suggesting comparable average squared error between predicted probabilities and observed outcomes. Post hoc Platt scaling significantly improved calibration for both models. The calibrated XGB model demonstrated a better balance of discrimination (AUC=0.822) and reliability (Brier=0.069, ECE=0.138), making it a reliable choice for clinical risk stratification, where both ranking and probability interpretation are essential. The reliability diagrams for the predictive models are displayed in Figure 2.

**Table 2:** Discrimination and calibration performance measures on the test set.

Models	AUC^a^	Brier score	ECE^b^	Calibration intercept	Calibration slope
LR^c^ model	0.799	0.181	0.362	–2.32	0.82
LR + Platt scaling	0.799	0.073	0.144	0.01	1.01
XGB^d^ model	0.822	0.175	0.323	–2.26	0.80
XGB + Platt scaling	0.822	0.069	0.138	0.31	1.14

^a^AUC: area under the receiver operating characteristic curve.

^b^ECE: expected calibration error.

^c^LR: logistic regression.

^d^XGB: extreme gradient boosting.

Table 3 summarizes the temporal validation results, including discrimination and multiple calibration indices (Brier score, ECE, calibration-in-the-large, and calibration slope). Overall, both models maintained strong discrimination in the temporal cohort. Post hoc calibration using Platt scaling improved calibration for both models, as reflected by reduced calibration error and calibration slopes closer to 1.0. For the calibrated XGB model, the calibration slope remained near one, and the calibration-in-the-large was relatively stable in the temporal cohort, suggesting limited temporal drift in calibration. In contrast, the calibrated LR model exhibited a larger change in the calibration intercept (from 0.01 to 1.02), consistent with a shift in baseline outcome risk over time and indicating that periodic recalibration may be more important for LR under the temporal distribution shift.

**Table 3:** Temporally validated models’ discrimination and calibration performance.

Models	AUC^a^	Brier score	ECE^b^	Calibration intercept	Calibration slope
Temporal LR^c^ model	0.823	0.166	0.346	–2.19	0.89
Temporal LR + Platt scaling	0.823	0.075	0.147	1.02	1.00
Temporal XGB^d^ model	0.842	0.162	0.306	–2.14	0.78
Temporal XGB + Platt scaling	0.842	0.069	0.140	6e-10	1.00

^a^AUC: area under the receiver operating characteristic curve.

^b^ECE: expected calibration error.

^c^LR: logistic regression.

^d^XGB: extreme gradient boosting.

### About DCA

**Figure 3:**
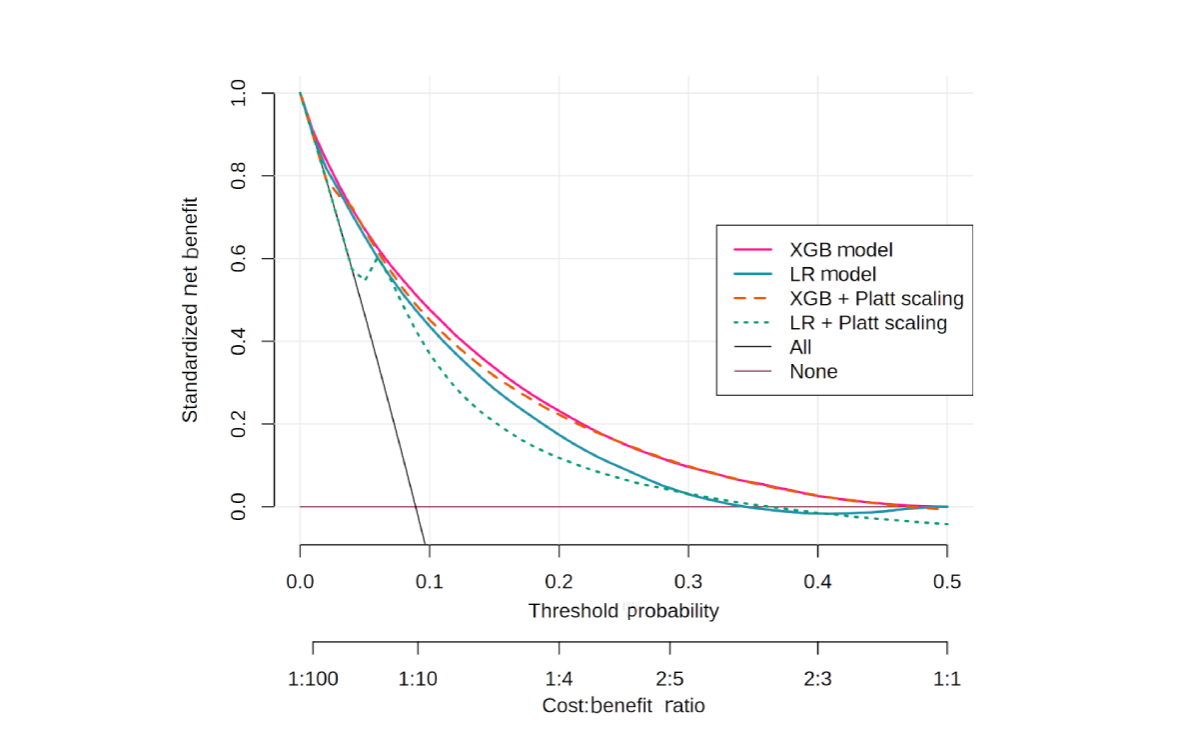
Decision curve analysis of XGB and LR models. LR: logistic regression; XGB: extreme gradient boosting.

Figure 3 presents the clinical utility of prediction models, showing standardized NB across a range of threshold probabilities compared to the default scenarios of “treat all” and “treat none.” The XGB and LR models demonstrated broadly similar clinical utility, with XGB consistently yielding slightly higher NB across all thresholds. At low thresholds (10%), both models achieved substantial benefit (NB ≈0.04), indicating a slight advantage over treating all (NB ≈–0.01) or none (NB ≈0). As the threshold increased, the NB of both models gradually declined. However, XGB maintained positive NB up to a threshold probability of approximately 0.5 (cost:benefit ratio ≈1:1), while LR converged toward the “treat none” line near 0.35 (cost:benefit ratio ≈2:5). For instance, at a clinically meaningful threshold of 20% (cost:benefit ratio ≈1:4), where missing a true DHD case is considered 4 times worse than providing an unnecessary intervention, the XGB model retained nearly 50% higher NB (0.020 vs 0.013) compared with LR. This suggests that XGB remains clinically useful even when decision-makers adopt more conservative intervention thresholds, when the cost of unnecessary intervention is comparable to the cost of missing a high-risk case, whereas LR provides meaningful benefit mainly at lower thresholds (0.05-0.35). Calibration using Platt produced only marginal changes, indicating improved probability reliability (Figure 2) but no substantial gain in clinical utility (Figure 3). More detailed results of the DCA analysis are provided in Multimedia Appendix 5.

### Fairness Analysis

**Figure 4:**
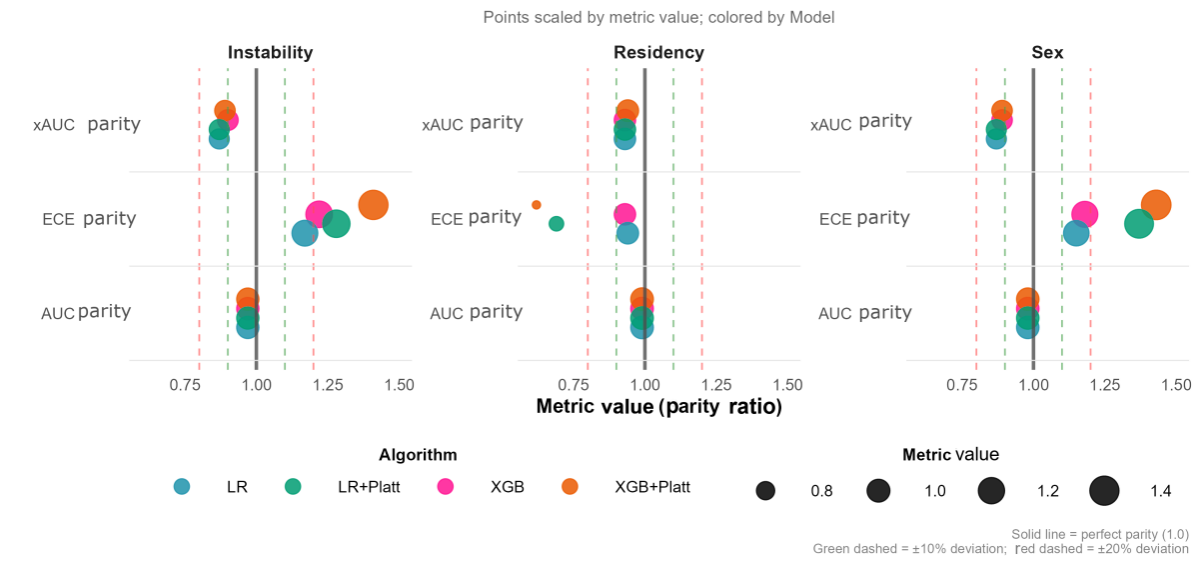
Comparing algorithmic fairness metrics across different predictive models and protected features. The size of each marker represents the magnitude of the fairness metric. The vertical dashed lines at 0.9-1.1 and 0.8-1.2 represent 10% and 20% deviation thresholds from perfect fairness (1.0). AUC: area under the receiver operating characteristic curve; ECE: expected calibration error; LR: logistic regression; xAUC: cross-group area under the curve; XGB: extreme gradient boosting.

Figure 4 illustrates threshold-free fairness metrics, specifically xAUC parity, ECE parity, and AUC parity, evaluated across 3 equity-seeking groups: females, rural residents, and highly marginalized populations characterized by residential instability. Each point represents a subgroup-specific fairness value, colored by model type and scaled according to its magnitude, with the ideal fairness benchmark of 1.0. Both models demonstrated strong within-group ranking fairness, with AUC parity values consistently approaching 1.0, and robust cross-group ranking fairness, reflected by xAUC parity values generally exceeding 0.9. In contrast, calibration fairness (ECE parity) revealed more substantial disparities, particularly for sex and residential instability, where values often fell outside the ±20% deviation threshold commonly used in the fairness literature [50]. Although Platt scaling improved global calibration performance, its uniform probability adjustment inadvertently magnified calibration differences between groups, thereby reducing ECE parity.

While most metrics remain within a satisfactory level of fairness (less than 20% deviation from perfect parity), prior work has shown that even modest deviations can compound into clinically meaningful inequities for vulnerable populations [21]. Achieving perfect fairness across all criteria simultaneously is inherently difficult due to known fairness-fairness and fairness-accuracy trade-offs [51,52]. To further improve algorithmic fairness and explore its impact on both fairness-fairness and fairness-accuracy trade-offs, we applied postprocessing bias mitigation. This allowed us to assess not only improvements in calibration fairness but also the interactions between fairness criteria and potential impacts on predictive performance.

**Figure 5:**
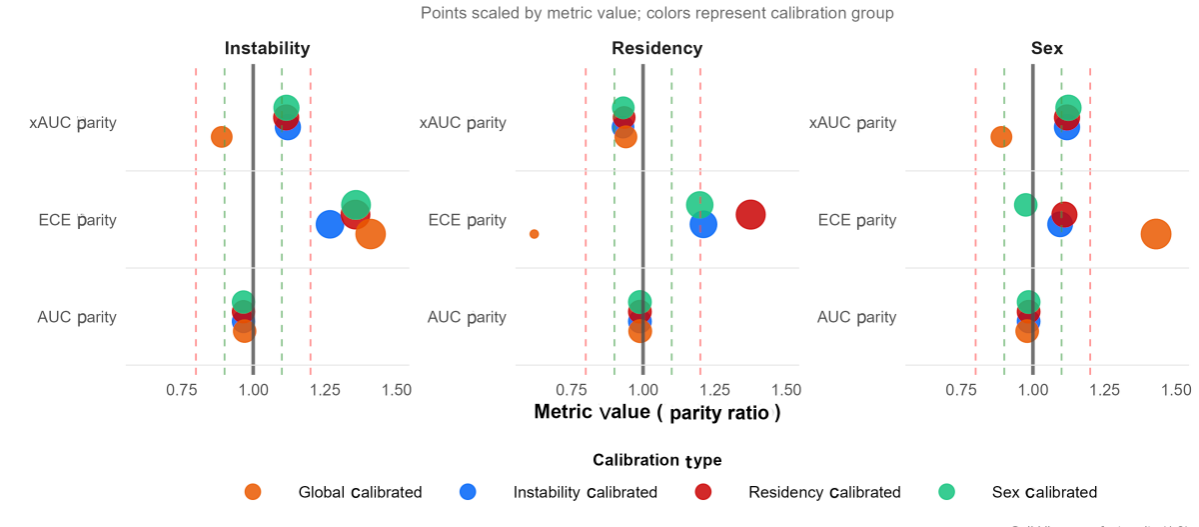
Comparison of group-based calibration for the XGB before and after post hoc bias mitigation. The size of each marker represents the magnitude of the fairness metric. The vertical dashed lines at 0.9-1.1 and 0.8-1.2 represent 10% and 20% deviation thresholds from perfect fairness (1.0). AUC: area under the receiver operating characteristic curve; ECE: expected calibration error; xAUC: cross-group area under the curve.

By implementing the postprocessing bias mitigation described in the section Fairness Assessment, we used group-stratified Platt scaling to enhance calibration fairness (ECE parity) in the XGB model (Figure 5). Among the approaches tested, stratification based on residential instability (depicted by the blue points in Figure 5) proved most effective, yielding substantial improvements in ECE parity across all 3 equity-seeking attributes when compared with global calibration (orange points). This strategy particularly improved calibration parity for sex, bringing ECE parity close to ideal levels (0.975) and well within the ±20% acceptability threshold, followed by moderate improvements in residency and smaller reductions in instability. However, deviations in the latter 2 groups remained slightly above the acceptable range (1.21-1.36). This indicates that residual calibration gaps persist for residency and residential instability even after post hoc mitigation, representing a limitation of group-stratified Platt scaling. Importantly, within-group ranking fairness (AUC parity) remained stable across all calibration schemes (0.966-0.989), confirming that probability rescaling preserved internal risk ordering. Cross-group ranking equity (xAUC parity) showed only minor fluctuations (eg, shifting from approximately 0.9 to 1.1 for instability and sex) yet consistently stayed within accepted bounds.

The findings highlight both the promise and limitations of targeted post hoc calibration. Specifically, group-aware recalibration, especially when anchored to the most imbalanced attribute, can significantly mitigate calibration disparities. However, achieving simultaneous improvements across all fairness dimensions remains challenging, reflecting the inherent fairness-fairness trade-off. Moreover, the selection of the stratification attribute introduces a fairness-target trade-off: enhancing calibration for 1 equity-seeking group may unintentionally diminish calibration performance for others. For example, sex-stratified calibration achieved near-perfect parity for sex but performed less favorably for residential instability. Conversely, calibration based on instability improved fairness across all groups, including residency, and in some cases outperformed residency-specific calibration. These patterns illustrate that fairness interventions interact in complex ways; that is, improvements in 1 demographic context can introduce miscalibration in others, highlighting the need for deliberate, context-sensitive prioritization when designing equitable clinical prediction models.

### Global Explainability

**Figure 6:**
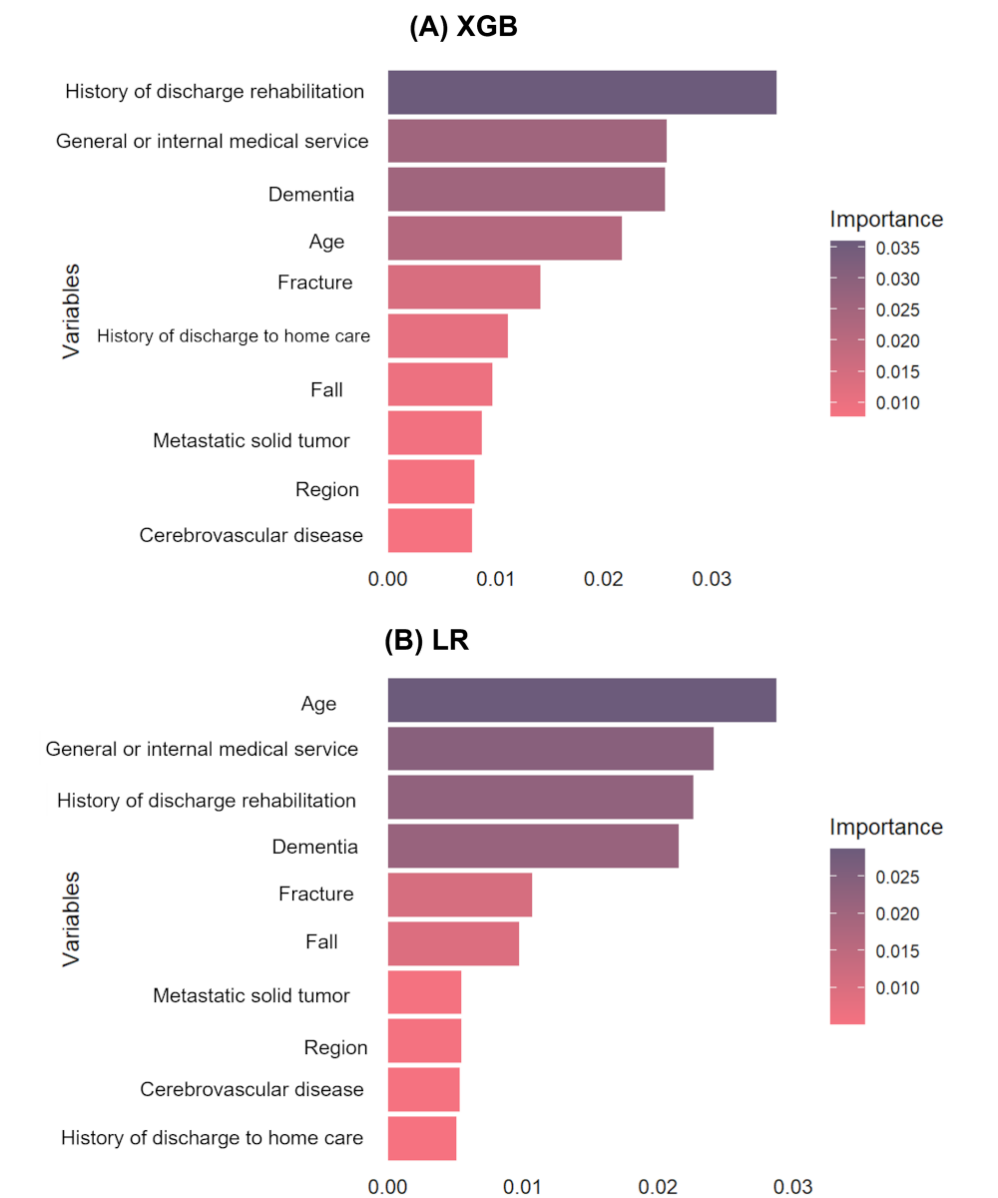
Top 10 most significant predictors by permutation feature importance scores. (A) Important features by XGB and (B) important features by LR. Hist: history; LR: logistic regression; Med: medical; Rehab: rehabilitation; XGB: extreme gradient boosting.

Figure 6 presents the top 10 most significant predictors, as determined by permutation feature importance scores. Both the XGB and LR models identified several key predictors in common, including the history of needing care support, dementia, fracture, rehabilitation care history, and age. Interestingly, while age was deemed the most important predictor in LR, it held a lower ranking in XGB, suggesting nonlinear models may more effectively capture interactions between variables, thereby reducing the standalone importance of age. Furthermore, the inclusion of regional variables among the top predictors suggests that DHD is not solely driven by patient-level clinical characteristics but is also influenced by geographic or system-level factors.

**Figure 7:**
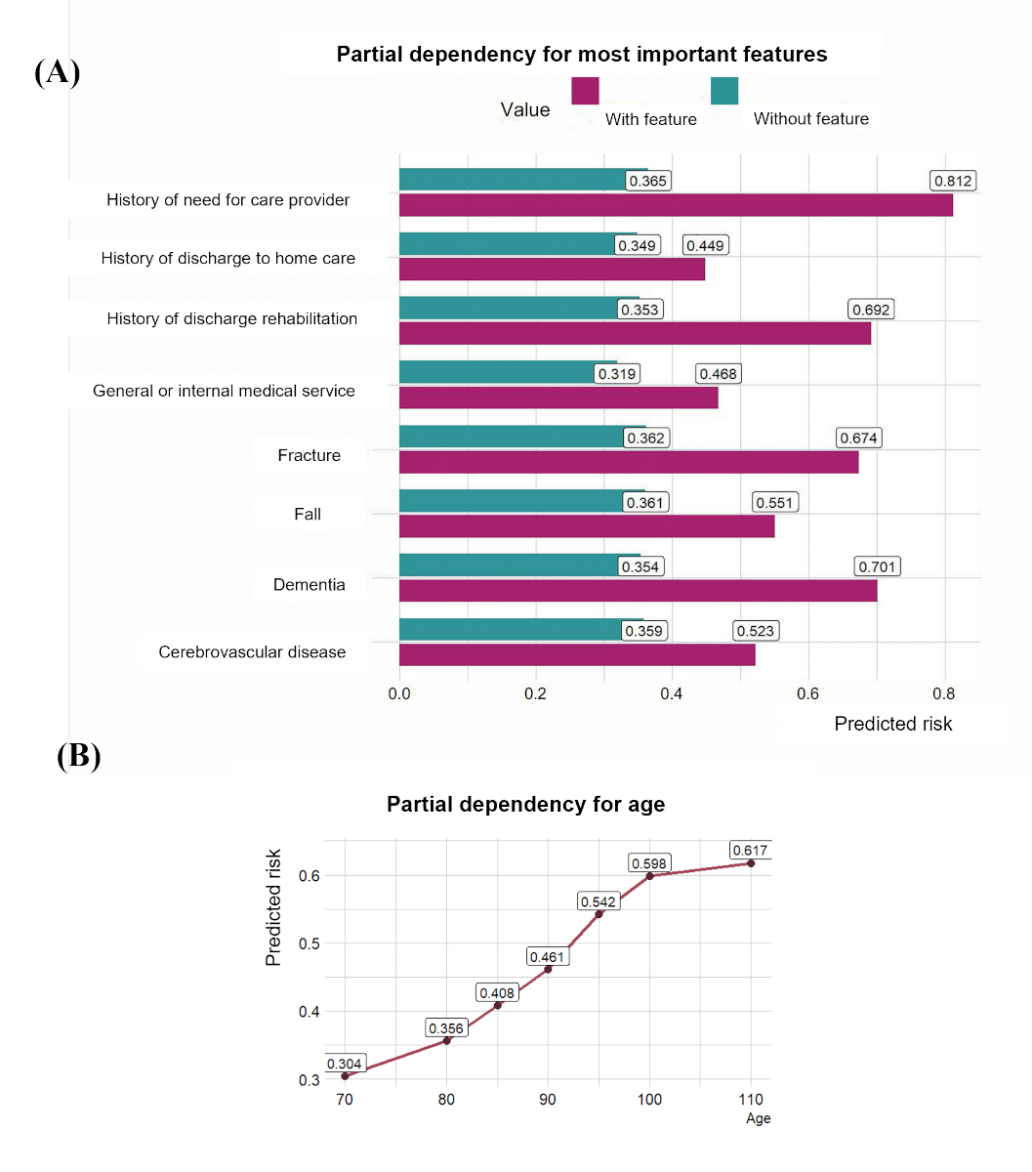
Exploring global explainability: partial dependency of top predictors. (A) Clinical factors, (B) demographic factors (age). Hist: history; Med: medical; Rehab: rehabilitation.

Due to XGB’s superior predictive and fairness performances compared to LR, we focused our subsequent analysis on this model for the explainability analysis. Figure 7 shows the PDP for these variables. This figure demonstrates how the predicted risk changes for each predictor when it changes from 0 (with the feature absent) to 1 (with the feature present). As can be seen, the history of need for care support, dementia, and history of discharge to rehabilitation care settings show the most significant changes in predicted risk. These changes are significant enough to raise predicted probabilities from below to above a 0.5 threshold, a commonly used decision point in classification tasks. For instance, patients with dementia exhibit predicted risks exceeding 0.7, underscoring their pivotal role in the risk of DHD. Age also demonstrates a consistent upward trend, with risk increasing steadily from 0.3 at age 70 years to over 0.6 by age 110 years, highlighting age as a critical predictor at the global level. Additionally, geographic factors appear to influence the predicted risk, with the Northern and Central regions of Ontario associated with the highest risks of delayed discharge (0.43 and 0.39, respectively). In contrast, the Eastern region indicates the lowest risk (0.33). This finding suggests potential geographic disparities in care or health system factors.

### Local Explainability

**Figure 8:**
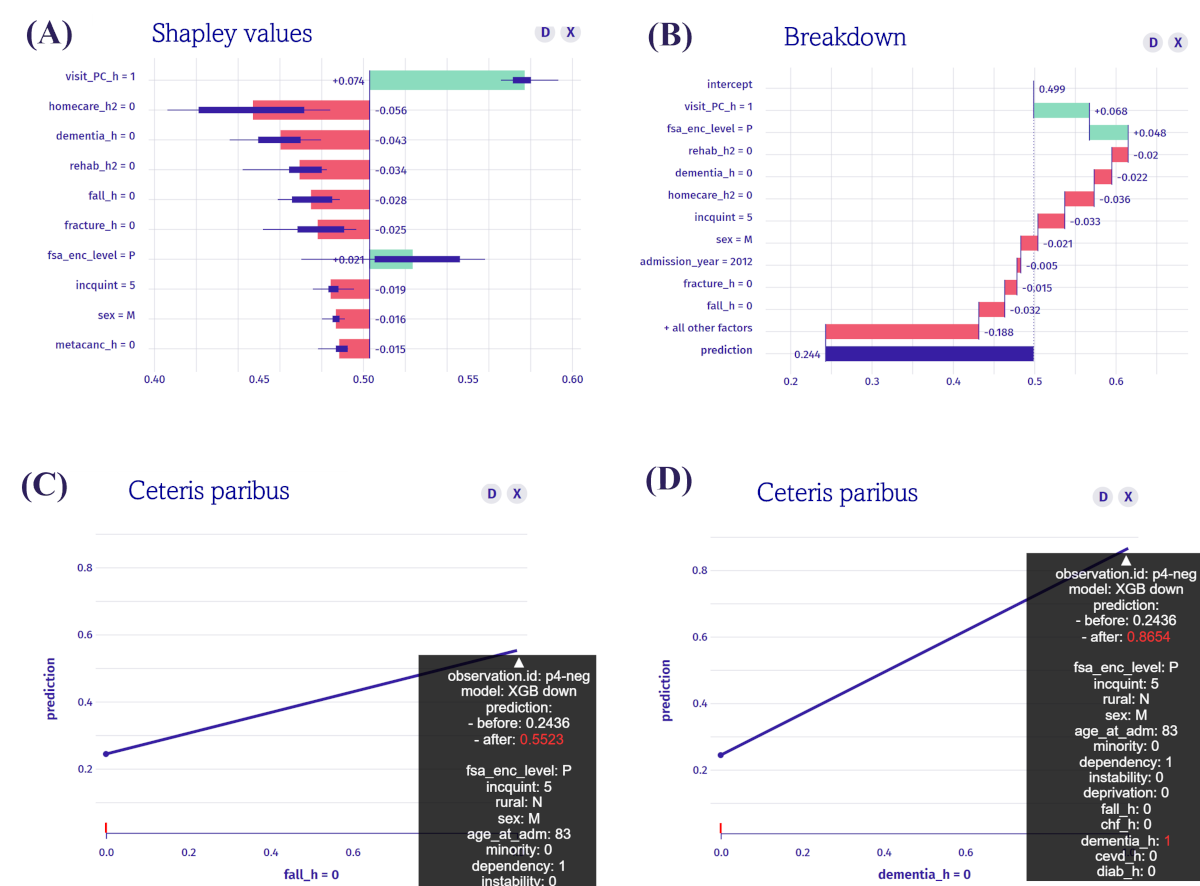
Local explainability panel for 1 deidentified patient: (A) SHAP values, (B) breakdown plot, (C) ceteris paribus (fall) indicates that introducing a fall history increases the predicted risk from 0.24 to 0.55, while holding other features constant, and (D) ceteris paribus (dementia) reveals a sharper effect, with the risk rising to 0.86 if dementia were present. SHAP: Shapley Additive Explanation; XGB: extreme gradient boosting.

To illustrate the practical utility of XAI for personalized care planning, we examined a deidentified patient with a predicted DHD risk of 0.24 (Figure 8). While this individual was classified as “low-risk” based on the overall model output, local explainability approaches, including SHAP values, breakdown plots, and ceteris paribus profiles, revealed that the prediction was highly sensitive to minor changes in specific clinical features. The SHAP and breakdown plots showed that the patient’s lack of fall history and dementia significantly lowered the predicted risk of DHD. Simulating the presence of either condition using ceteris paribus profiles, while holding all other variables constant, significantly increased the predicted risk from 0.24 to 0.55 if the same person had a history of falls, and to 0.86 if the person had dementia. This example illustrates how individual-level explainability can uncover a phenomenon we define as “clinical risk instability,” a condition in which a patient’s health status appears stable until a specific risk factor shifts, resulting in a significant increase in risk. Such underlying vulnerabilities and their critical insights can be achieved through local explainability, whereas global explanations cannot provide such capability.

**Figure 9:**
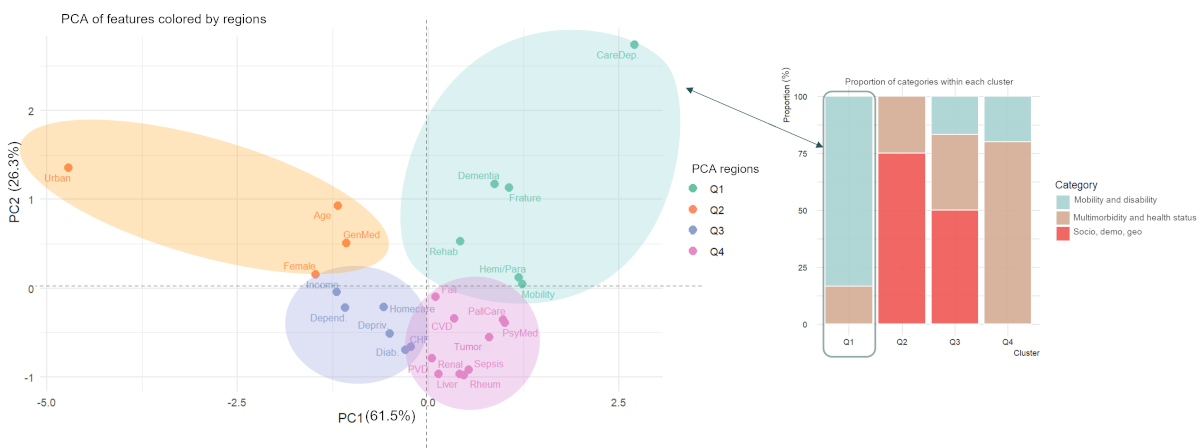
Local interpretations of feature contributions: feature breakdown, clustering, and category distribution. CareDep: history of need for care support; CHF: chronic heart failure; CVD: cerebrovascular disease; Depend: dependency; Depriv: deprivation; Diab: diabetes; GenMed: general medicine service; Hemi/Para: hemiplegia or paraplegia; PallCare: palliative care history; PCA: principal component analysis; PsyMed: psychiatry service; PVD: peripheral vascular disease; Rehab: history of discharge to rehabilitation center; Rheum: rheumatoid; Socio-Demo-Geo: socioeconomic, demographic, and geographic.

As explained in the Methods section, we also used PCA to cluster features based on their contributions, frequencies, and rankings from SHAP and breakdown explanations for high-risk patients. The resulting biplot is displayed in Figure 9, accounting for 87.8% of the data variance, with 61.5% attributed to PC1 and 26.3% to PC2. PC1 primarily captures the trade-off between high contributions and rankings versus lower frequencies, reflecting features that exert strong but less ubiquitous influence on predictions.

In contrast, PC2 emphasizes high contributions paired with frequent appearances, irrespective of ranking, highlighting features that are both impactful and commonly relevant across high-risk cases:

*PC*1=0.47×*contribution*+0.65×*rank*–0.60×*percentage*

*PC*2=0.85×*contribution*–0.15×*rank*+0.51×*percentage*

The coefficients of the variables indicate the magnitude of their influence in the principal component, while their signs reveal whether they exert a positive or negative influence [49]. We categorized the resulting PCA space into 4 regions (Q1-Q4) based on the signs of the coefficients for each feature along the PC1 and PC2 axes. Region Q1, depicted by green points, is characterized by high values on both components and primarily comprises features related to mobility and disability, which account for over 75% of its attributes. The remaining features in Q1 are related to multimorbidity and health status, with no contribution from sociodemographic or geographic factors. This pattern indicates that the loss of independence and the presence of chronic conditions are pivotal in predicting DHD risk. The bar chart confirms that mobility-related issues are significant contributors to DHD.

This PCA-derived clustering provides deeper interpretive value by grouping features with similar profiles, facilitating a holistic understanding beyond individual explanations. For instance, the dominance of mobility and disability in Q1 suggests that interventions targeting physical function could mitigate risks for a substantial portion of high-risk patients, offering policymakers actionable insights to prioritize community-based rehabilitation services that enhance or maintain mobility. Such services, delivered by physiotherapists, occupational therapists, and kinesiologists, include strength and balance training, support for activities of daily living, and personalized exercise programs, and have demonstrated effectiveness in preventing functional decline and alleviating acute care burdens [53]. The history of the need for care support consistently ranked as the most significant contributor in the mobility and disability category and as one of the most impactful features. In the category of multimorbidity, dementia emerged as the most significant factor. Overall, these results reinforce the utility of PCA clustering in distilling complex local data into strategic patterns, bridging microlevel insights with macrolevel policy implications for more equitable and efficient health care delivery. Feature-level PCA outputs, including each predictor’s PC1 and PC2 values, quadrant assignment (Q1-Q4), and predefined conceptual categories, are provided in Multimedia Appendix 4 to support transparency and reproducibility of the clustering interpretation.

## Discussion

### Principal Findings

Our study emphasizes the potential of ML to accurately predict DHD by using extensive longitudinal administrative health data. The XGB model outperformed LR in both predictive accuracy and fairness, supporting the effectiveness of advanced ML techniques in identifying patients at risk for DHD. This predictive capability facilitates proactive interventions, helping to alleviate care bottlenecks and enhance discharge planning.

Explainability analyses revealed that high-risk predictions were predominantly driven by mobility and functional impairments, as well as cognitive impairment factors (eg, dementia). These findings are aligned with previous literature that consistently highlights dementia as one of the most significant risk factors for delayed discharge [54]. PCA clustering of local explanations further highlighted the predominance of mobility and disability features in the high-impact quadrant, accounting for over 75% of attributes there, which shows the critical importance of functional and cognitive declines on high-risk predictions and informs targeted preventive strategies. Local explainability results further shed light on how individual features influence predictions, clarifying what might otherwise remain a “black box.” For instance, partial dependency analysis revealed substantial risk shifts associated with dementia and the need for care support, emphasizing their importance in clinical decision-making. We also observed regional patterns in predicted risk, consistent with studies on geographic disparities with respect to clinical outcomes [55]. By connecting these insights to actionable interventions, such as implementing earlier discharge planning for high-risk groups and conducting routine fear-of-falling screening in primary and community care to trigger fall-prevention referrals [56,57], and ensuring access to postacute rehabilitation for individuals with dementia and those at the risk of falls who are often excluded under current eligibility criteria [58], our study contributes to the growing body of work aiming to make ML tools both interpretable and impactful in real-world health care settings.

Fairness emerged as another important aspect of our analysis. Our results indicate that the models performed fairly across important subgroups, such as sex, rural-urban residency, and residential instability, supporting the broader goal in health care ML to prevent inequities. We highlighted the complex trade-offs that arise not only between fairness and accuracy but also among various aspects of fairness themselves, emphasizing the value of involving decision-makers in the model development process, as resolving these tensions often requires contextual knowledge and consideration of policy goals. This aligns with principles highlighted in a *New England Journal of Medicine* study, specifically regarding the ethical use of AI in health care [59].

### Clinical, Managerial, and Policy Implications

DHDs pose a significant challenge to health care systems, straining hospital capacity and compromising patient care. By identifying patients at risk of delayed discharge in advance, our findings enable health administrators to proactively allocate necessary supportive services, such as home care referrals, rehabilitative and therapeutic care, and community support, where they are most needed. This approach promotes a more efficient and proactive health care system, ultimately reducing unnecessary admissions that may lead to prolonged hospital stays. Second, understanding the factors that consistently contribute to an elevated risk of delayed discharge can reveal systemic deficiencies. If patients with specific mobility or cognitive impairments are frequently categorized as high-risk, it may indicate underinvestment in and insufficient publicly funded community-based rehabilitation services. Recognizing these deficiencies can help direct policy initiatives and funding toward addressing them, thereby enhancing system capacity over time. Third, by synthesizing individual-level insights, health system planners can monitor trends within specific population subgroups. Regions consistently associated with higher risk or patient cohorts facing distinct challenges can be targeted through specialized programs, infrastructure investments, or policy reforms. Fourth, leveraging local XAI analyses, such as ceteris paribus and SHAP, to detect clinical risk instability (where a patient’s seemingly stable condition rapidly shifts to a high-risk state) empowers clinicians to initiate anticipatory interventions, including cognitive screening, home safety evaluations, or pre-emptive referrals to community services.

At a systems level, this highlights the value of integrating XAI tools not only to explain retrospective predictions but also to inform real-time, forward-looking care strategies that align with the principles of personalized and preventive medicine. Finally, with a clearer understanding of how various factors interact to hinder timely discharge, decision-makers can implement strategic interventions, such as early geriatric assessments, specialized dementia care pathways, and improved coordination with community care providers to prevent hospitalization delays. Instead of responding to existing backlogs, policymakers can take proactive measures to enhance the quality and continuity of care.

### Limitations

First, the predictive models are developed using retrospective cohort data, which limits control over data collection and can introduce selection bias. Second, our predictive models only focus on older delayed-discharge patients; therefore, their applicability to other populations is limited. Third, our data may lack confounders, such as social determinants of health (eg, individual-level income, individual-level education, race, housing support, caregiver availability, and capacity), the severity of cognitive or physical impairments, and caregiver support levels. Thus, predictive model performance may improve by incorporating these risk factors. Fourth, PDPs summarize average marginal associations between predictors and model predictions. However, when predictors are correlated, PDP estimates can be influenced by the joint distribution of features and may implicitly rely on extrapolation into combinations of covariate values that are uncommon or unsupported in the observed data, complicating causal or purely marginal interpretation [46,60]. Future work could use accumulated local effects profiles as a robustness check, as accumulated local effects are less sensitive to feature dependence and can provide more stable global effect summaries under correlation. Finally, model performance was evaluated using a large held-out test set and an independent temporally separated cohort, providing an assessment of both internal performance and temporal generalizability. Given the size of the test set, performance estimates from a single evaluation are expected to be stable under the law of large numbers; however, this approach does not provide explicit uncertainty estimates (eg, CIs). Consequently, while XGB consistently demonstrated higher discrimination and improved calibration relative to LR across both the random hold-out and temporal validation settings, we caution against overinterpreting performance differences. Future work could strengthen comparative inference and quantify performance variability through repeated cross-validated evaluation or nested cross-validation, particularly when the objective is to establish statistically robust superiority between competing models.

### Conclusions

Our study used an integrated approach that develops predictive models, ensures fairness, and incorporates XAI, offering a forward-looking perspective on the ongoing challenge of delayed discharge. By identifying at-risk patients early and promoting equitable outcomes while illuminating the factors driving our predictions, we established a more robust foundation for prevention-oriented policies. By focusing on accuracy, fairness, and explainability, this study advances the development of ML models that are not only accurate but also ethical and interpretable. These triple considerations are critical for fostering trust in AI-driven decision-making in health care and improving patient outcomes. This enables health system leaders, policymakers, clinicians, and community partners to implement evidence-based interventions that address the root causes of DHD. Ultimately, this enhances their ability to manage care, reduce delays, optimize resource allocation, and improve the quality and equity of care for older adults at risk of delayed discharge.

## Data Availability

We are unable to provide a minimal dataset for this study due to privacy, legal, and ethical restrictions, as well as prescribed entity designations. All data used in this study are securely housed at ICES, Ontario, Canada, in coded form and are subject to their privacy, legal, prescribed entity designations, and ethical governance. While legal data-sharing agreements between ICES and data providers (eg, health care organizations and government) prohibit ICES from making the dataset publicly available, access may be granted to those who meet prespecified criteria for confidential access. A high-level script skeleton and pseudocode outlining the full analysis pipeline are publicly available [61].

## Appendix

Multimedia Appendix 1 Overview of data sources and cohort characteristics.

Multimedia Appendix 2 Data preparation.

Multimedia Appendix 3 Technical and package information.

Multimedia Appendix 4 Mapping features.

Multimedia Appendix 5 Decision curve analysis.

## Abbreviations

AI: artificial intelligence
AUC: area under the receiver operating characteristic curve
DCA: decision curve analysis
DHD: delayed hospital discharge
ECE: expected calibration error
HiREB: Hamilton Integrated Research Ethics Board
ICES: Institute for Clinical Evaluative Sciences
LR: logistic regression
LTC: long-term care
ML: machine learning
NB: net benefit
ON-Marg: Ontario Marginalization Index
PDP: partial dependence plot
PCA: principal component analysis
SHAP: Shapley Additive Explanations
XAI: explainable artificial intelligence
xAUC: cross-group area under the curve
XGB: extreme gradient boosting
