# Supplementary material for: Responsible AI for Predicting Delayed Hospital Discharge Among Older Adults: Development and Evaluation Study for Balancing Accuracy, Equity, and Explainability
Source: JMIR Med Inform. 2026 Apr 13;14:e83244. doi: 10.2196/83244 (PMC13122139; doi:10.2196/83244)
Supplement: Multimedia Appendix 1 [file medinform_v14i1e83244_app1.docx]

## **Data Description**

This study primarily utilized the Discharge Abstract Database (DAD), a national administrative database maintained by the Canadian Institute for Health Information (CIHI). The DAD collects standardized administrative and clinical data on all hospital separations—including discharges, transfers, and deaths—from acute care facilities throughout Canada, except for Quebec. In Ontario, all hospitals that provide acute or surgical day care services are mandated to report to CIHI through the DAD [1].

Data collection follows a standardized process. When a patient is admitted, their health record is created using the hospital’s admission, discharge, and transfer system. Upon discharge, trained health information professionals abstract key clinical and administrative information from the patient’s chart and submit electronic records to CIHI. CIHI performs automated file integrity testing and data validation, assigns case-mix and resource-intensity weights, and generates operational reports that are reviewed by the submitting facilities. These facilities then address any data quality issues and resubmit corrections as necessary. Once the data has been validated and accepted, it is incorporated into the DAD and made available for analysis through CIHI’s secure analytical environment, such as ICES [2].

## **Descriptive Analysis Results**

Table S1 describes the clinical variables chosen for model development.

**Table S1**- Clinical Characteristics

| Characteristic† | Overall,  N = 8,900,589*^1^* | Without Delayed Discharge  N = 8,107,313*^1^* | With Delayed Discharge  N = 793,276*^1^* |
| --- | --- | --- | --- |
| Cancer (any malignancy) | 1,741,537 (20%) | 1,600,613 (20%) | 140,924 (18%) |
| Care-provider dependency | 98,205 (1.1%) | 58,970 (0.7%) | 39,235 (4.9%) |
| Cerebrovascular | 676,147 (7.6%) | 551,530 (6.8%) | 124,617 (16%) |
| CHF | 1,310,672 (15%) | 1,125,700 (14%) | 184,972 (23%) |
| Chronic pulmonary | 1,098,741 (12%) | 961,127 (12%) | 137,614 (17%) |
| Dementia | 594,291 (6.7%) | 426,289 (5.3%) | 168,002 (21%) |
| Developmental Disability | 2,598 (0.029%) | 2,200 (0.027%) | 398 (0.050%) |
| Diabetes | 1,473,649 (17%) | 1,322,468 (16%) | 151,181 (19%) |
| Diabetes with Com. | 1,347,962 (15%) | 1,179,457 (15%) | 168,505 (21%) |
| Fall | 758,015 (8.5%) | 574,059 (7.1%) | 183,956 (23%) |
| Fracture | 524,243 (5.9%) | 380,497 (4.7%) | 143,746 (18%) |
| General/Internal Medicine Service | 3,709,044 (42%) | 3,171,057 (39%) | 537,987 (68%) |
| Hemiplegia/paraplegia | 110,235 (1.2%) | 81,582 (1.0%) | 28,653 (3.6%) |
| Homecare History | 2,384,131 (27%) | 2,037,454 (25%) | 346,677 (44%) |
| Metastatic-solid tumour | 473,332 (5.3%) | 414,376 (5.1%) | 58,956 (7.4%) |
| Mild liver disease | 128,906 (1.4%) | 113,337 (1.4%) | 15,569 (2.0%) |
| Mobility | 85,077 (1.0%) | 62,985 (0.8%) | 22,092 (2.8%) |
| Mod. severe liver dis. | 80,330 (0.9%) | 71,759 (0.9%) | 8,571 (1.1%) |
| Myocardial infarction (MI) | 899,115 (10%) | 802,435 (9.9%) | 96,680 (12%) |
| Palliative Care History | 113,893 (1.3%) | 92,473 (1.1%) | 21,420 (2.7%) |
| Peptic Ulcer | 233,456 (2.6%) | 210,621 (2.6%) | 22,835 (2.9%) |
| Peripheral vascular | 425,084 (4.8%) | 372,707 (4.6%) | 52,377 (6.6%) |
| Physical Disability | 1,597,320 (18%) | 1,370,999 (17%) | 226,321 (29%) |
| Psychiatry Service | 36,394 (0.4%) | 30,379 (0.4%) | 6,015 (0.8%) |
| Rehabilitation Care History | 614,000 (6.9%) | 454,572 (5.6%) | 159,428 (20%) |
| Renal Disease | 691,416 (7.8%) | 592,025 (7.3%) | 99,391 (13%) |
| Rheumatoid | 115,416 (1.3%) | 98,810 (1.2%) | 16,606 (2.1%) |
| Sensory Disability | 1,749,456 (20%) | 1,661,874 (20%) | 87,582 (11%) |
| Sepsis | 227,752 (2.6%) | 194,199 (2.4%) | 33,553 (4.2%) |
| *^1^* n (%); *^2^* CI = Confidence Interval | | | |
| *Note: The characteristics are arranged in alphabetical order.*  *† All the clinical predictors were coded as binary variables reflecting the history of presence or absence of each comorbidity or clinical condition (e.g., history present = 1, history absent = 0) based on the two-year look-back period.* | | | |

**Outcome Prevalence Summary: Train vs. Test**

**Table S2-** The prevalence of the 90-day DHD in train/test and by equity-seeking subgroup

| Factor | Data | ALC Prevalence (%) |
| --- | --- | --- |
| Sex | Test | F: 10.42, M: 7.47 |
| Sex | Train | F: 10.32, M: 7.56 |
| Sex | All | F: 10.33, M: 7.55 |
| Instability | Test | 0: 8.05, 1: 11.15 |
| Instability | Train | 0: 8.09, 1: 10.97 |
| Instability | All | 0: 8.09, 1: 10.99 |
| Rural or Urban | Test | N: 9.15, Y: 7.69 |
| Rural or Urban | Train | N: 9.14, Y: 7.68 |
| Rural or Urban | All | N: 9.14, Y: 7.68 |

**References:**

[1] Canadian Institute for Health Information. Discharge Abstract Database (DAD) Metadata 2025. https://www.cihi.ca/en/discharge-abstract-database-dad-metadata.

[2] Canadian Institute for Health Information. How to Monitor Your DAD and/or NACRS Data Submissions. Ottawa, ON: Canadian Institute for Health Information; 2023.
