# Supplementary material for: Responsible AI for Predicting Delayed Hospital Discharge Among Older Adults: Development and Evaluation Study for Balancing Accuracy, Equity, and Explainability
Source: JMIR Med Inform. 2026 Apr 13;14:e83244. doi: 10.2196/83244 (PMC13122139; doi:10.2196/83244)
Supplement: Multimedia Appendix 2 [file medinform_v14i1e83244_app2.docx]

1. **Historical-based (Look-back) for clinical variables**

We implemented a 2-year look-back period from the index acute care admission to capture comorbidities and other clinical variables using structured administrative data available at ICES. All clinical variables were derived from coded datasets—primarily the Discharge Abstract Database (DAD)—with diagnostic information recorded using International Statistical Classification of Diseases and Related Health Problems, 10^th^ Revision (ICD-10) codes. For each patient, DAD contains up to 25 diagnosis fields per admission, where these fields were queried across the two years before the index admission to identify whether specific conditions or clinical measures were present during that time. For chronic conditions, we focused on those defined in the Charlson Comorbidity Index [1], using validated ICD-10 mappings and implemented through the *comorbidity* library in R. If a comorbidity appeared in any admission record within the two-year look-back, the patient was flagged as having a history of that condition. This approach captures chronic diseases that persist across encounters and mitigates potential under-coding in any single admission. For clinical variables not included in the Charlson Index, we used specific ICD-10 codes to define and create those variables. No manual chart abstraction or unstructured text processing was performed; all data were obtained from structured, coded administrative sources.

**Table S1**- ICD-10 codes for disability and mobility-related conditions

| **Variable** | **ICD10- codes** |
| --- | --- |
| Fall | W00-W19: Slipping, tripping, stumbling and falls^[[1]](#footnote-1)^ |
| Mobility | R26.2: Difficulty in walking, not elsewhere classified  R26.8: Other and unspecified abnormalities of gait and mobility  Z99.3: Dependence on wheelchair  ***Reference:*** [2] |
| Problems related to care-provider dependency | Z74.0: Reduced mobility, bedfast, chairfast  Z74.1: Need for assistance with personal care  Z74.2: Need for assistance at home and no other household member able to render care  Z74.3: Need for continuous supervision  Z74.8: Other problems related to care-provider dependency  Z74.9: Problem related to care-provider dependency, unspecified  ***Reference:***[2] |
| Physical Disability | **Congenital anomalies:**  Q67.5, Q66, Q67.6, Q67.7, Q67.8, E34.3, E23.0, Q01.9, Q02, Q03, Q04, Q06, Q07.8, Q07.9, G90.1, Q75, Q76, Q77, Q78, Q79, Q72, Q73, Q74, Q71, Q05, Q70  **Musculoskeletal disorders:**  E22.0, M45, M46, M86.3, M86.6, M50.0, M50.2, M50.3, M50.4, M50.5, M50.6, M50.7, M50.8, M50.9, M51.0, M51.2, M51.9, M22.4, M23.2, M23.3, M23.4, M23.5, M23.8, M23.9, M15, M16, M17, M18, M19, M42, M91, M92, M93, M87, M80, M35.3, M05, M06, M47  **Neurological disorders:**  G80, G90, G40, G81, G60, G11, G32.8, G57, G58, G35, G71, G72, G70, G54, G55, G36, G37, G95, G24, G24.7, G24.8, G24.9, G24.4, G24.5, G24.6, G24.7, G24.8, G24.9, G25, G82, G83, G61, G62, G63, G31.8, G20, G21, I69, B91, G12  **Permanent injuries:**  S02.0, S02.1, S02.3, S02.7, S02.8, S02.9, S06.1, S06.2, S06.3, S06.4, S06.5, S06.6, S06.7, S06.8, S06.9, S07, T02.0, T90.5, S77, S87, S97, T04.1, T04.4, T04.5, T04.6, T04.7, T90.8, Z99.3, Z99.8, S32.4, S32.8, S32.9, T34.1, T34.2, T34.3, T34.4, T34.5, T34.6, T34.7, T34.8, S98.3, S98.8, S98.9, Z89.0, Z89.1, Z89.2, Z89.3, Z89.4, Z89.5, Z89.6, Z89.7, Z89.8, Z89.9  ***Reference:*** [3] |
| Sensory Disability | **Hearing impairments:**  H90, H91.3, H91.8, H91.9, Q16.0, Q16.1, Q16.3, Q16.4, Q16.5, Q16.6, Q16.7, Q16.8, Q16.9  **Vision impairments:**  H54, H25, H26, H30, H31, Q11.1, Q11.2, Q13.1, Q13.3, Q13.8, Q15.0, H44, H20.1, H47.6, H40, H42, H55, E10.31, E10.32, E10.33, E10.34, E10.35, E11.31, E11.32, E11.33, E11.34, E11.35, H34, H35, H36  ***Reference:*** [3] |
| Developmental Disability | **Autism spectrum disorder:**  F84.0, F84.1, F84.3, F84.4, F84.5, F84.6, F84.7, F84.8, F84.9  **Other Developmental Disability:**  Q86.0, F70, F71, F72, F73, F78, F79  Q90, Q91, Q92.0, Q92.1, Q92.2, Q92.3, Q92.4, Q92.5, Q92.7, Q92.8, Q92.9  Q93, Q97.1, Q99.2, Q99.8  Q85.1, Q86.1, Q87.1, Q87.23, Q87.31, Q87.8  ***Reference:*** [3] |

1. **Forward-looking (Look-ahead) for outcome variable**

To determine the look-ahead period, we evaluated each patient visit by looking 90 days ahead of the visit date. During this 90-day window, we assessed whether the patient experienced a delayed discharge. This approach ensures that for every visit, we systematically capture the occurrence of delayed discharge within the defined time frame. If a delayed discharge was observed during the look-ahead period, the variable (e.g., DHD =1) was set to indicate its presence. To ensure the robustness of our findings, sensitivity analyses were conducted using alternative timeframes of 180 and 365 days.

1. **Outcome sensitivity across timeframes (90, 180, 365)**

**Table S2**- Sensitivity analysis of outcome measure across different timeframes

| Variables | DHD -90d | DHD -180d | DHD -365d |
| --- | --- | --- | --- |
| Male | 0.812 (0.808-0.816) | 0.825 (0.821-0.829) | 0.841 (0.837-0.845) |
| Age | 1.056 (1.052-1.060) | 1.055 (1.056-1.057) | 1.055 (1.056-1.057) |
| Rural vs. Urban | 0.874 (0.867-0.881) | 0.873 (0.867-0.879) | 0.873 (0.867-0.879) |
| Ethnic concentration | 0.879 (0.872-0.886) | 0.872 (0.866-0.878) | 0.864 (0.858-0.870) |
| Dependency | 0.975 (0.969-0.980) | 0.973 (0.968-0.977) | 0.973 (0.968-0.977) |
| Residential Instability | 1.157 (1.150-1.164) | 1.162 (1.156-1.169) | 1.170 (1.164-1.176) |
| Material Deprivation | 1.069 (1.062-1.077) | 1.071 (1.064-1.078) | 1.071 (1.064-1.078) |
| Income-Q1 (lowest) | Ref. | Ref. | Ref. |
| Income-Q2 | 0.948 (0.940-0.955) | 0.947 (0.940-0.954) | 0.943 (0.936-0.950) |
| Income-Q3 | 0.940 (0.931-0.948) | 0.938 (0.930-0.946) | 0.929 (0.922-0.937) |
| Income-Q4 | 0.910 (0.901-0.918) | 0.911 (0.902-0.919) | 0.905 (0.897-0.912) |
| Income-Q5 (highest) | 0.855 (0.847-0.864) | 0.853 (0.845-0.861) | 0.848 (0.841-0.856) |
| Fall | 2.809 (2.791-2.827) | 2.643 (2.626-2.659) | 2.444 (2.427-2.455) |
| CHF | 1.271 (1.262-1.279) | 1.297 (1.289-1.305) | 1.306 (1.299-1.314) |
| Dementia | 3.037 (3.016-3.057) | 2.867 (2.848-2.886) | 2.629 (2.612-2.645) |
| Cerebrovascular | 1.818 (1.804-1.832) | 1.753 (1.740-1.766) | 1.673 (1.661-1.684) |
| Diabetes | 1.057 (1.051-1.065) | 1.077 (1.070-1.084) | 1.098 (1.092-1.105) |
| Diabetes with Com. | 1.272 (1.263-1.281) | 1.291 (1.282-1.299) | 1.309 (1.301-1.317) |
| Sepsis | 1.252 (1.236-1.268) | 1.219 (1.204-1.234) | 1.161 (1.148-1.175) |
| Peripheral vascular | 1.270 (1.257-1.283) | 1.281 (1.268-1.293) | 1.268 (1.257-1.279) |
| Chronic pulmonary | 1.263 (1.254-1.272) | 1.306 (1.298-1.315) | 1.353 (1.345-1.361) |
| Rheumatoid | 1.486 (1.460-1.513) | 1.523 (1.498-1.549) | 1.574 (1.550-1.599) |
| Peptic ulcer | 1.017 (1.002-1.032) | 1.033 (1.019-1.047) | 1.061 (1.048-1.075) |
| Mild liver disease | 1.461 (1.430-1.492) | 1.500 (1.471-1.530) | 1.526 (1.498-1.554) |
| Mod. severe liver dis. | 1.103 (1.072-1.134) | 1.139 (1.110-1.169) | 1.157 (1.130-1.185) |
| Renal disease | 1.271 (1.261-1.281) | 1.296 (1.287-1.306) | 1.320 (1.311-1.329) |
| Cancer (any malignancy) | 0.923 (0.916-0.930) | 0.964 (0.957-0.970) | 0.994 (0.988-1.001) |
| Metastatic-solid tumour | 2.175 (2.151-2.198) | 2.031 (2.011-2.052) | 1.803 (1.786-1.821) |
| Hemiplegia/paraplegia | 2.182 (2.149-2.218) | 2.097 (2.065-2.129) | 1.970 (1.940-1.999) |
| MI | 0.962 (0.955-0.970) | 0.963 (0.956-0.971) | 0.961 (0.955-0.968) |
| Hist. Discharge to Palliative | 1.800 (1.770-1.830) | 1.709 (1.682-1.736) | 1.600 (1.576-1.625) |
| Hist. Discharge to Rehabilitation | 2.43 (2.42-2.44) | 2.35 (2.34-2.36) | 2.24 (2.23-2.26) |
| Hist. Discharge to Homecare | 1.22 (1.21-1.23) | 1.29 (1.28-1.30) | 1.36 (1.35-1.36) |
| Fracture | 1.47 (1.46-1.48) | 1.42 (1.42-1.44) | 1.35 (1.34-1.36) |
| Mobility | 1.45 (1.43-1.48) | 1.45 (1.43-1.48) | 1.45 (1.43-1.48) |
| Hist. of need for care support | 2.83 (2.79-2.87) | 2.68 (2.65-2.72) | 2.52 (2.48-2.55) |
| Hist. main patient service: |  |  |  |
| General Medicine | 1.75 (1.74-1.76) | 1.73 (1.72-1.74) | 1.67 (1.66-1.67) |
| Psychiatry | 1.49 (1.45-1.54) | 1.52 (1.48-1.57) | 1.56 (1.52-1.61) |
| FSA-K (Eastern) | Ref. | Ref. | Ref. |
| FSA-L (Central) | 1.331 (1.321-1.341) | 1.325 (1.316-1.335) | 1.327 (1.318-1.336) |
| FSA-M (Metropolitan) | 1.155 (1.144-1.165) | 1.156 (1.146-1.166) | 1.165 (1.156-1.175) |
| FSA-N (Southwestern) | 1.130 (1.121-1.140) | 1.130 (1.121-1.139) | 1.138 (1.130-1.146) |
| FSA-P (Northern) | 1.562 (1.547-1.577) | 1.591 (1.577-1.606) | 1.627 (1.614-1.641) |
| Developmental Disability | 1.85 (1.66-2.05) | 1.72 (1.55-1.91) | 1.72 (1.55-1.91) |
| Sensory Disability | 0.48 (0.47-0.49) | 0.51 (0.52-0.53) | 0.51 (0.52-0.53) |
| Physical Disability | 1.96 (1.95-1.97) | 1.88 (1.87-1.89) | 1.88 (1.87-1.89) |

**References:**

[1] Deyo RA, Cherkin DC, Ciol MA. Adapting a clinical comorbidity index for use with ICD-9-CM administrative databases. Journal of Clinical Epidemiology 1992;45:613–9.

[2] Gilbert T, Neuburger J, Kraindler J, Keeble E, Smith P, Ariti C, et al. Development and validation of a Hospital Frailty Risk Score focusing on older people in acute care settings using electronic hospital records: an observational study. The Lancet 2018;391:1775–82.

[3] Brown H, Saeed G, Tarasoff L, Proulx L, Welsh K, Fung K, et al. Equity and inclusion in pregnancy care: report on the pregnancy outcomes and health care experiences of people with disabilities in Ontario. Toronto, ON: ICES; 2024.

1. https://www.icd10data.com/ICD10CM/Codes/V00-Y99/W00-W19 [↑](#footnote-ref-1)
