# Supplementary material for: Responsible AI for Predicting Delayed Hospital Discharge Among Older Adults: Development and Evaluation Study for Balancing Accuracy, Equity, and Explainability
Source: JMIR Med Inform. 2026 Apr 13;14:e83244. doi: 10.2196/83244 (PMC13122139; doi:10.2196/83244)
Supplement: Multimedia Appendix 3 [file medinform_v14i1e83244_app3.docx]

**Table S1.** R packages used in the study.

| Package | Purpose or Application | Reference |
| --- | --- | --- |
| tidyverse | Data cleaning and visualization | [1] |
| caret | Model development, training, and tuning | [2] |
| rmda | Decision curve analysis (DCA) | [3] |
| fairness | Fairness metrics and bias assessment | [4] |
| DALEX | Model explainability | [5] |
| modelStudio | Interactive dashboard for explainability results | [6] |
| isotone | Calibration | [7] |

**References:**

[1] Wickham H, Averick M, Bryan J, Chang W, McGowan LD, François R, et al. Welcome to the Tidyverse. Journal of Open Source Software 2019;4:1686.

[2] Kuhn M. The caret package. 2011.

[3] Brown M. Risk Model Decision Analysis 2018. https://mdbrown.github.io/rmda/ (accessed November 3, 2025).

[4] Kozodoi N, Varga TV. fairness: Algorithmic Fairness Metrics 2021.

[5] Biecek P. DALEX: Explainers for complex predictive models in R. Journal of Machine Learning Research 2018;19:1–5.

[6] Baniecki H, Biecek P. modelStudio: Interactive Studio with Explanations for ML Predictive Models. JOSS 2019;4:1798. https://doi.org/10.21105/joss.01798.

[7] Mair P, De Leeuw J, Hornik K, Mair MP. Package ‘isotone’ 2015.
