# Supplementary material for: Responsible AI for Predicting Delayed Hospital Discharge Among Older Adults: Development and Evaluation Study for Balancing Accuracy, Equity, and Explainability
Source: JMIR Med Inform. 2026 Apr 13;14:e83244. doi: 10.2196/83244 (PMC13122139; doi:10.2196/83244)
Supplement: Multimedia Appendix 4 [file medinform_v14i1e83244_app4.docx]

**Table S1**. Feature mapping across socio-demographic, health, and mobility categories.

| Feature (short name) | PC1 | PC2 | Quadrant | Category |
| --- | --- | --- | --- | --- |
| History of Need for Care Support (CareDep.) | 2.70 | 2.74 | Q1 | Mobility & Disability |
| Frature | 1.06 | 1.13 | Q1 | Mobility & Disability |
| Dementia | 0.88 | 1.17 | Q1 | Multimorbidity & Health Status |
| Hemiplegia/paraplegia (Hemi/Para) | 1.19 | 0.12 | Q1 | Mobility & Disability |
| Mobility | 1.24 | 0.04 | Q1 | Mobility & Disability |
| History of Discharge: Rehab. Care (Rehab) | 0.42 | 0.53 | Q1 | Mobility & Disability |
| Residency: Urban | -4.72 | 1.35 | Q2 | Socio-Demo-Geo |
| Age (mean) | -1.18 | 0.93 | Q2 | Socio-Demo-Geo |
| Sex: Female | -1.47 | 0.15 | Q2 | Socio-Demo-Geo |
| General Medicine Service (GenMed) | -1.07 | 0.51 | Q2 | Multimorbidity & Health Status |
| Dependecny (Depend.) | -1.09 | -0.22 | Q3 | Socio-Demo-Geo |
| Income Level (Income) | -1.20 | -0.05 | Q3 | Socio-Demo-Geo |
| Material Deprivation (Depriv.) | -0.51 | -0.51 | Q3 | Socio-Demo-Geo |
| Diabetes (Diab.) | -0.29 | -0.69 | Q3 | Multimorbidity & Health Status |
| CHF | -0.23 | -0.66 | Q3 | Multimorbidity & Health Status |
| History of Discharge: Homecare (Homecare) | -0.58 | -0.21 | Q3 | Mobility & Disability |
| Rheumatoid (Rheum) | 0.47 | -0.98 | Q4 | Multimorbidity & Health Status |
| Sepsis | 0.53 | -0.92 | Q4 | Multimorbidity & Health Status |
| Psychiatry Service (PsyMed) | 1.00 | -0.39 | Q4 | Multimorbidity & Health Status |
| Mild liver disease (Liver) | 0.42 | -0.96 | Q4 | Multimorbidity & Health Status |
| Metastatic-solid tumour (Tumour) | 0.80 | -0.55 | Q4 | Multimorbidity & Health Status |
| Palliative Care History (PallCare) | 0.98 | -0.36 | Q4 | Mobility & Disability |
| PVD | 0.14 | -0.96 | Q4 | Multimorbidity & Health Status |
| Renal disease (Renal) | 0.05 | -0.79 | Q4 | Multimorbidity & Health Status |
| Cerebrovascular (CVD) | 0.34 | -0.34 | Q4 | Multimorbidity & Health Status |
| Fall | 0.10 | -0.09 | Q4 | Mobility & Disability |
