# Supplementary material for: Responsible AI for Predicting Delayed Hospital Discharge Among Older Adults: Development and Evaluation Study for Balancing Accuracy, Equity, and Explainability
Source: JMIR Med Inform. 2026 Apr 13;14:e83244. doi: 10.2196/83244 (PMC13122139; doi:10.2196/83244)
Supplement: Multimedia Appendix 5 [file medinform_v14i1e83244_app5.docx]

**Table S1.** Net benefit (NB) and standardized N=net benefit (sNB) at selected thresholds.

| **Model** | **Threshold Probability** | **Net Benefit (NB)** | **Standardized Net Benefit (sNB)** |
| --- | --- | --- | --- |
| XGB | 0.05 | 0.0599 | 0.6712 |
| XGB | 0.1 | 0.0423 | 0.4746 |
| XGB | 0.2 | 0.0203 | 0.2274 |
| XGB | 0.3 | 0.0084 | 0.0944 |
| XGB | 0.4 | 0.0024 | 0.0285 |
| XGB | 0.5 | 0.0002 | 0.0021 |
| LR | 0.05 | 0.0539 | 0.6526 |
| LR | 0.1 | 0.0389 | 0.4358 |
| LR | 0.2 | 0.0138 | 0.1526 |
| LR | 0.3 | 0.0017 | 0.0299 |
| LR | 0.4 | -0.0014 | -0.0147 |
| LR | 0.5 | 0 | 0.0002 |
| All | 0.05 | -0.0104 | -0.1347 |
| All | 0.1 | -0.012 | -0.1534 |
| All | 0.2 | -0.1385 | -1.5531 |
| All | 0.3 | -0.3012 | -3.3768 |
| All | 0.4 | -0.5180 | -5.8084 |
| All | 0.5 | -0.8216 | -9.2125 |
| None | 0.05 | 0 | 0 |
| None | 0.1 | 0 | 0 |
| None | 0.2 | 0 | 0 |
| None | 0.3 | 0 | 0 |
| None | 0.4 | 0 | 0 |
| None | 0.5 | 0 | 0 |
